# SOX7 is down-regulated in lung cancer

**DOI:** 10.1186/1756-9966-32-17

**Published:** 2013-04-04

**Authors:** Takahide Hayano, Manoj Garg, Dong Yin, Makoto Sudo, Norihiko Kawamata, Shuo Shi, Wenwen Chien, Ling-wen Ding, Geraldine Leong, Seiichi Mori, Dong Xie, Patrick Tan, H Phillip Koeffler

**Affiliations:** 1 Genomic Oncology Programme, Cancer Science Institute of Singapore, NUS, Singapore, 14 Medical Drive, #12-01, Singapore 117599, Singapore; 2 Department of Hematology and Oncology, Cedars-Sinai Medical Center, Los Angeles, CA, USA; Cedar- Sinai Medical Center, 8700 Beverly Boulevard, Davis 5068, Los Angeles, CA, 90048, USA; 3 Key Laboratory of Nutrition and Metabolism, Institute for Nutritional Sciences, Shanghai Institutes for Biological Sciences, Chinese Academy of Sciences and Graduate School of Chinese Academy of Sciences, Shanghai, 200031, China; 4 Division of Cancer Genomics, The Cancer Institute of Japanese Foundation for Cancer Research, Tokyo; 3-8-31 Ariake, Koto-ward, Tokyo Post-code 135-8550, Tokyo, Japan; 5 Duke-NUS affiliation to Cancer and Stem Cell Biology, Duke-NUS Graduate Medical School, 8 College Road, Singapore, 169857, Singapore; 6 National University Cancer Institute, Singapore, National University Hospital, Singapore, 1E Kent Ridge Road, NUHS Tower Block, Level 7, Singapore, 119228, Singapore

**Keywords:** CNAG, SNP-Chip, Lung cancer, SOX7, Promoter methylation

## Abstract

**Background:**

SOX7 is a transcription factor belonging to the SOX family. Its role in lung cancer is unknown.

**Methods:**

In this study, whole genomic copy number analysis was performed on a series of non-small cell lung cancer (NSCLC) cell lines and samples from individuals with epidermal growth factor receptor (EGFR) mutations using a SNP-Chip platform. SOX7 was measured in NSCLC samples and cell lines, and forced expressed in one of these lines.

**Results:**

A notable surprise was that the numerous copy number (CN) changes observed in samples of Asian, non-smoking EGFR mutant NSCLC were nearly the same as those CN alterations seen in a large collection of NSCLC from The Cancer Genome Atlas which is presumably composed of predominantly Caucasians who often smoked. However, four regions had CN changes fairly unique to the Asian EGFR mutant group. We also examined CN changes in NSCLC lines. The SOX7 gene was homozygously deleted in one (HCC2935) of 10 NSCLC cell lines and heterozygously deleted in two other NSCLC lines. Expression of SOX7 was significantly downregulated in NSCLC cell lines (8/10, 80%) and a large collection of NSCLC samples compared to matched normal lung (57/62, 92%, p= 0.0006). Forced-expression of SOX7 in NSCLC cell lines markedly reduced their cell growth and enhanced their apoptosis.

**Conclusion:**

These data suggest that SOX7 is a novel tumor suppressor gene silenced in the majority of NSCLC samples.

## Introduction

Lung cancer is the leading cause of cancer-related death in the world. If surgery is inadequate, further therapy is rarely curative. Understanding the genomic abnormalities in this disease affords the opportunity to identify new therapeutic targets. An example is the use of Gefitinib for patients whose non-small cell lung cancer (NSCLC) has an epidermal growth factor receptor (EGFR) mutation in either exon 19 or 21.

SOX7 is a member of the SOX (SRY-related high mobility group box) transcription factors [1]. This protein, together with SOX17 and SOX18, comprises the SOX F subgroup [2] and helps mediate various developmental processes including a role in the regulation of hematopoiesis [3], cardiogenesis [4], vasculogenesis [5,6], endoderm differentiation [7] and myogenesis [8]. Recently, SOX7 has been proposed to function as a tumor suppressor in colorectal and prostate cancers [9,10]. We provide evidence that SOX7 behaves as a tumor suppressor in lung tissue and its expression is either low or silenced in the majority of lung cancers.

## Materials and methods

### Cell lines and tissue samples

Ten human lung cancer cell lines (H23, H460, H820, H1299, H1975, HCC827, HCC2279, HCC2935, HCC4006, PC14) were cultured in RPMI medium with 10% FBS and kept in a humidified atmosphere of 5% CO_2_. After IRB consent, total DNA and RNA of normal and cancerous lung tissues were obtained from the National University of Singapore (NUH-NUS Tissue Repository). Also, sixty-two pairs of primary NSCLCs and their corresponding adjacent normal tissues, which were at least 5 cm away from the cancer, were obtained from NSCLC patients treated at Shanghai Chest Hospital (Shanghai, China), after their written informed consent. None of the patients received radio-chemotherapy prior to obtaining the tissues. Lung cancer cells stably expressing either GFP or SOX7 were generated by transducing them with PLKO.1 lentiviral vector system (Sigma). Briefly, cells were transduced with lentiviral vectors (SOX7 or GFP) at an MOI of 25 with 5 ug/ml polybrene added for 6 h. Twenty-four hours post-transduction, stable cells were selected using 1ug/ml puromycin for 2-3 weeks.

### High-density single nucleotide polymorphism-array analysis

Genomic DNA from NSCLC cells were subjected to GeneChip Human mapping (1000 K array for the EGFR mutant lung cancer samples and 250 K array for the NSCLC cell lines). Both total and allelic-specific copy numbers (CN) were determined using CNAG software [11,12].

### Quantitative real-time polymerase chain reaction

Real-time reverse transcriptase polymerase chain reaction (RT-PCR) was performed using Maxima^®^ First Strand cDNA Synthesis Kit for RT-qPCR (Fermentas) according to the manufacturer’s protocol. The expression level of SOX7 mRNA in the samples was determined by quantitative real-time PCR (7500 Fast Real-Time PCR System, Applied Biosystems) using KAPA™ SYBR^®^ FAST qPCR Kit Master Mix (2X) Universal (Kapa Biosystems). Levels of β-actin mRNA were used as an internal control. The delta threshold value (DCt) was calculated from the given threshold (Ct) value by the formula DCt = (Ct SOX7 – Ct β-actin) for each sample.

### Western blotting

NSCLC cells were lysed with ProteoJET™ Mammalian Cell Lysis Reagent (Fermentas). Immunoblotting was performed using either anti-SOX7 antibody (Sigma, HPA009065) or anti-β-actin antibody (Sigma, AC-15) and either secondary anti-Rabbit IgG antibody (GE Healthcare, NA934) or anti-murine IgG antibody (GE Healthcare, NA931), respectively. SOX7 or β-actin bands were detected using Pierce^®^ Fast Western Blot Kit, SuperSignal^®^ West Femto Substrate (Thermo SCIENTIFIC) and SuperSignal^®^ West Pico Chemiluminescent Substrate (Thermo SCIENTIFIC), respectively.

### Bisulfite sequencing

Genomic DNA was modified by sodium bisulfite using the CpGenome™ Turbo Bisulfite Modification Kit (MILLIPORE). The following PCR primers were used for bisulfite-modified genomic DNA [10]:

Region (-687 to -440): 5’-TTAATTAGGTGGTTGAGAATTAGAA and 5’-TAACCATAAACCCCTCAAAACA

Region (-71 to +251): 5’-TTTTGGAGAGTTATTGGAGGA and 5’-CCTTAACCCAAACCATAAAAA

PCR products were cloned into either the pGEM-T or pGEM-T easy vector (Promega), and at least four clones from each sample were sequenced.

### Methylation specific PCR (MSP) assay

Primers specific for the unmethylated (U) and methylated (M) sequences were designed by using Meth Primer [13]. Primers sequences are as follows:

MSP-U (-683 to -493): 5'-TAGGTGGTTGAGAATTAGAATGAT G and 5'-CTTTCAAAAATAACCAAACTTCAAC

MSP-M (-683 to 493): 5'-TTAGGTGGTTGAGAATTAGAACGAC and 5'-TCGAAAATAACCGAACTTCGA

MSP-U (+192 to +321): 5'-ATAAGGGTTTTGAGAGTTGTATTTG and 5'-ACTCACCCAACATCTTACTAAACTCA

MSP-M (+192 to +321): 5'-ATAAGGGTTTCGAGAGTCGTATTC and 5'-TCACCCAACATCTTACTAAACTCG

### MTT assay

H23 and H1975 cells were seeded at 5 × 10^3^ per well in 96-well plates. H1299 cells were seeded at 1.5 × 10^3^ per well in 96-well plates. MTT reagents were added to each well, and absorbance was measured according to the manufacturer’s instructions (Promega).

### Cell cycle analysis by flow cytometry

2x10^6^ cells stably expressing either SOX7 or GFP were seeded into 6-well plates for 24 h. Cells were harvested and washed twice with cold phosphate-buffered saline (PBS) and fixed in 75% ethanol (precooled at -20°C) for 24 h at 4°C. The fixed cells were washed twice with 2 ml of cold PBS. Cells then were stained with 500 ul of propidium iodide (PI) staining solution (50 ug/ml PI, 0.1%Triton X-100, 200 mg/ml DNase-free RNase in PBS) for 30 min at room temperature in the dark. Ten thousand events per sample were acquired using a LSR-II flow cytometer (Becton-Dickinson, San Jose, CA, USA), and the percentage of cells in G_0_/G_1_, S, G_2_/M and Sub-G_2_/M phases of the cell cycle were determined using FACS DIVA software (Becton-Dickinson).

### Annexin V and propidium iodide (Annexin V–PI) staining apoptosis test

4 × 10^5^ cells were seeded into each well of a 6-well plate for 48 h. The staining was carried out according to the instructions provided by the manufacturer of PE Annexin V Apoptosis Detection Kit I, BD Pharmingen (BD Biosciences, USA). Briefly, cells were washed with PBS, suspended in 1X binding buffer and then added with annexin-V APC and propidium iodide (PI) for 15 min. The samples were then analyzed by LSR-II flow cytometer (Becton-Dickinson, San Jose, CA, USA).

## Results

### Whole genomic copy number analysis using high resolution SNP-Chips in NSCLC samples and cell lines

**Figure 1:**
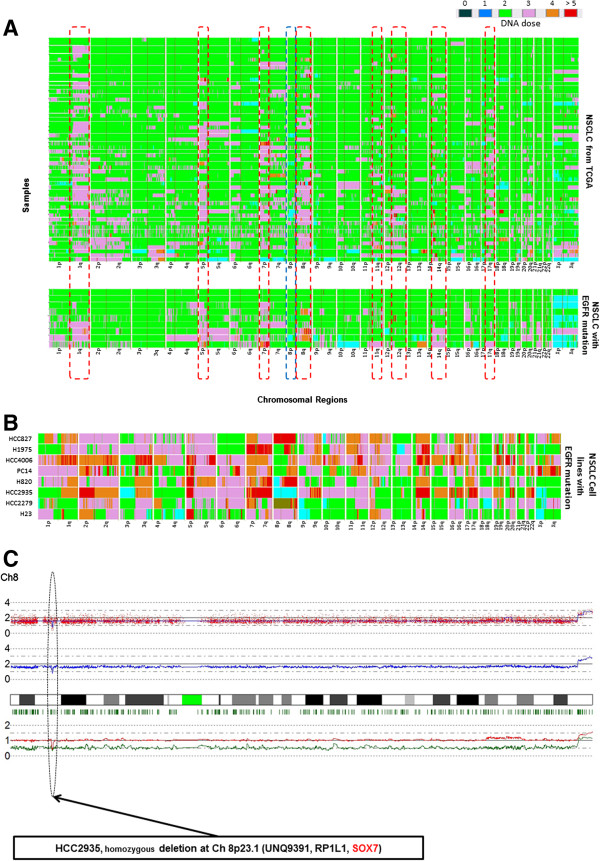
**Whole genome copy number analysis using high resolution SNP**-**Chips.** (**A**, **B**) Heat map of DNA copy numbers found in the p and q arms of the chromosomes (horizontal axis) of 56 NSCLC samples from the TCGA data base [top panel, Figure 1A], 9 NSCLC patient samples with EGFR mutations [bottom panel, Figure 1A] and 8 NSCLC cell lines with EGFR mutations [Figure 1B] (vertical axis). DNA copy numbers are indicated by colors (black, blue, green, pink, orange and red are 0, 1, 2, 3, 4 and ≥5 copies, respectively). Common copy number gain regions are emphasized by red dotted rectangles. Common copy number loss region is emphasized by blue dotted rectangle. (**C**) At chromosome 8p23.1, a homozygous deletion of SOX7 occurs in the HCC2935 NSCLC cell line. Red dots show raw data. Blue line denotes total gene dosage by CNAG; level 2 indicates diploid (2N) amount of DNA. Sample is mostly hemizygous. Green small vertical bars immediately under the chromosome display heterozygous SNP sites. The bottom lines (Red and Green) denote allele-specific gene dosage (one line indicates gene dosage of the maternal allele, and the other indicates gene dosage of the paternal allele). Sample shows that chromosome 8 is hemizygously deleted except at 8p23.1 where the second allele is also lost in a small region resulting in homozygous deletion of the UNQ9391, RP1L1 and the SOX7 genes.

Initially, genomic alterations were examined in a small sample set of Asians with NSCLC with EGFR mutations. Nine clinical NSCLC samples with EGFR mutation were analyzed for copy number aberrations (CNA) using a high-resolution SNP-Chip microarray platform (Affymetrix). The alterations of the CNA in these mutant EGFR samples were compared to 56 NSCLC samples from The Cancer Genome Atlas (TCGA) data base. The mutational status of EGFR in these 56 NSCLC samples is not available; but because most of the patients are Caucasians from the USA, the EGFR in the NSCLC probably is mutated in less than 7% of these cases [14]. The overall genomic profiles of NSCLC were highly similar when comparing our samples having a mutant EGFR and the samples in the TCGA data base (Figure 1A; Table 1). This is consistent with our earlier study where we reported this observation across a larger cohort [15]. For example, 78% (7/9) and 75% (42/56) of samples of both cohorts had gain at 5p13.2, and 67% (6/9) and 73% (41/56) of samples had gain at 8q24.12-24.3, respectively. Nevertheless, several CNAs were associated with the EGFR mutation-positive NSCLC samples (Table 2). For example, 89% (8/9) of our EGFR mutant tumors versus 27% (15/56) of the TCGA samples had CN gain at 1p36.31-36.32; also, 56% (5/9) of our EGFR mutant samples versus 11% (6/56) of the TCGA samples had gain at 19q12. Clearly, too few EGFR mutant samples were analyzed to perform statistical analysis. We also did SNP analysis on 8 EGFR mutant NSCLC cell lines. These cell lines frequently had CN gain throughout much of each chromosome (Figure 1B). Loss of CN in the NSCLC samples and cell lines was infrequent, occurring slightly more often at 6q22.3-27, 8p, and 9p21.3 (Figure 1A, B; Tables 1, 2).

**Table 1:** Common copy number genomic alterations in NSCLC found in two cohorts: TCGA and EGFR mutant, non-smoking Asians

**Region of Chromosome**	**Candidate target genes**
Gain of 1q21.1q-24.2	Large fragment
Gain of 5p13.2	SKP2
Gain of 7p11.2	EGFR
Gain of 8q24.3	PTP4A3
Gain of 8q24.21	MYC, PVT1
Gain of 8q24.12	MTBP
Loss of 8p23.1	UNQ9391, RP1|1, **SOX7**
Gain of 11q13.2-13.3	CYCLIN D1, FGF3, FGF4, FGF19
Gain 12q14.2	TBK1, RASSF3
Gain 12q14.3	HMGA2
Gain of 12q13.3-14.1	CDK4
Gain of 12q12.1	KRAS
Gain of 12q11.21	DDX11
Gain 14q13.3	PAX9
Gain of 17q12	Her2
Gain of 17q25.3	TK1, BIRC5

Common genomic alterations found in both NSCLC samples with EGFR mutations (9 samples) and those from the TCGA data base [56 samples, probably these rarely have an EGFR mutation (Zhou et al. [14])].

**Table 2:** Copy number genomic alterations that predominant in NSCLC from non-smoking Asians with mutant EGFR compared to TCGA database

**Region of Chromosome**	**NSCLC with mutant EGFR (n=8)**	**NSCLC from TCGA data base (n=56)**	**Potential target gene(s)**
Gain of 1p36.32-36.31	8/9(89)	15/56(27%)	AJAP1
Gain of Ch2p	Fewer alteration	More alterations	Large fragment
Gain of Ch3q	Fewer alteration	More alterations	Large fragment
Loss of 6q22.3-27	Fewer alteration	More alterations	Large fragment
Loss of 9p21.3	1/9(11%)	19/56(34%)	p14,p15,p16
Gain of 15q23-26.3	0/9(0%)	10/56(18%)	Large fragment
Gain of 19q12	6/9(70%)	6/56(11%)	Cyclin E1
Gain of 20q11.21	0/9(0%)	26/56(46%)	BCL2L1, TPX2, MYLK2, DUSP15

Ratio of genomic alterations in NSCLC samples with EGFR mutations (9 samples) and 56 NSCLC samples from the TCGA data base. [Most samples from TCGA are from Caucasians and thus we assume <7% will have EGFR mutation as previously noted (Zhou et al. [14])].

Cell lines enhance the opportunity to discover homozygous deletions because they are not contaminated with normal cells. A homozygous deletion often marks the position of a tumor suppressor gene that may be deleterious for either development or progression of cancer. A small homozygous deletion at 8p23.1 was found in one (HCC2935) of 10 NSCLC cell lines. The SOX7 was located in this small homologously deleted region together with 2 other genes (UNQ9391 and RP1L1) (Figure 1C; Table 1).

### Expression of SOX7 in NSCLC

**Figure 2:**
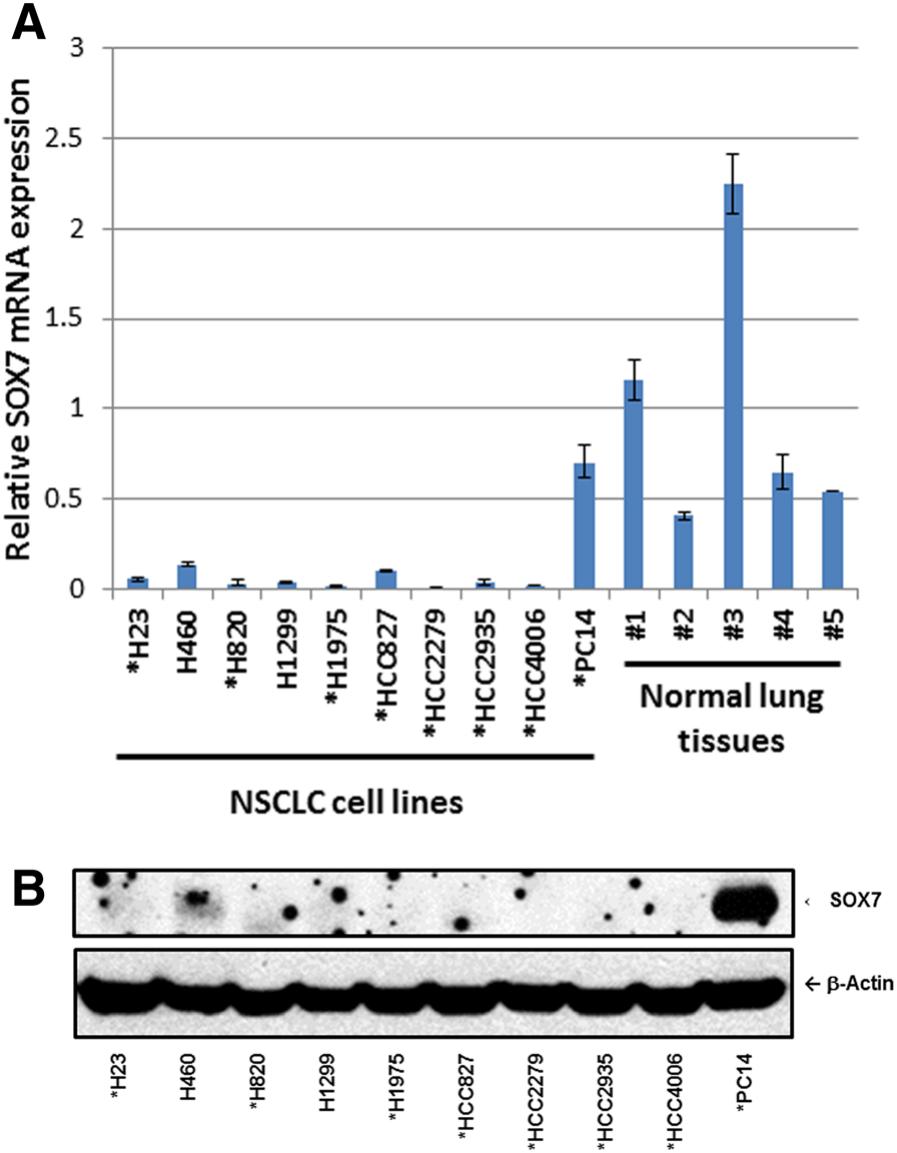
**Down**-**regulation of SOX7 in NSCLC cells.** (**A**) Real-time reverse transcription-PCR measurement of expression of SOX7 mRNA in 10 NSCLC cell lines and 5 normal lung samples. Relative expression level 1.0 represents the mean expression of the 5 normal lung tissues. (**B**) Western blot analysis of SOX7 expression of the same 10 NSCLC cell lines. β-actin is used as the loading control. (*) denotes EGFR mutated cell lines.

Expression of SOX7 gene was examined initially in 10 human NSCLC cell lines using quantitative RT-PCR (qRT-PCR). Compared with the average SOX7 mRNA level (arbitrary level 1) of five normal lung tissues, nine of the 10 cell lines exhibited extremely low levels of SOX7 mRNA (mean level was 12% of the average found in the normal lung tissues) (Figure 2A). In addition, SOX7 protein expression was only weakly detected in two (H460 and PC14) of these 10 NSCLC cell lines (Figure 2B).

**Figure 3:**
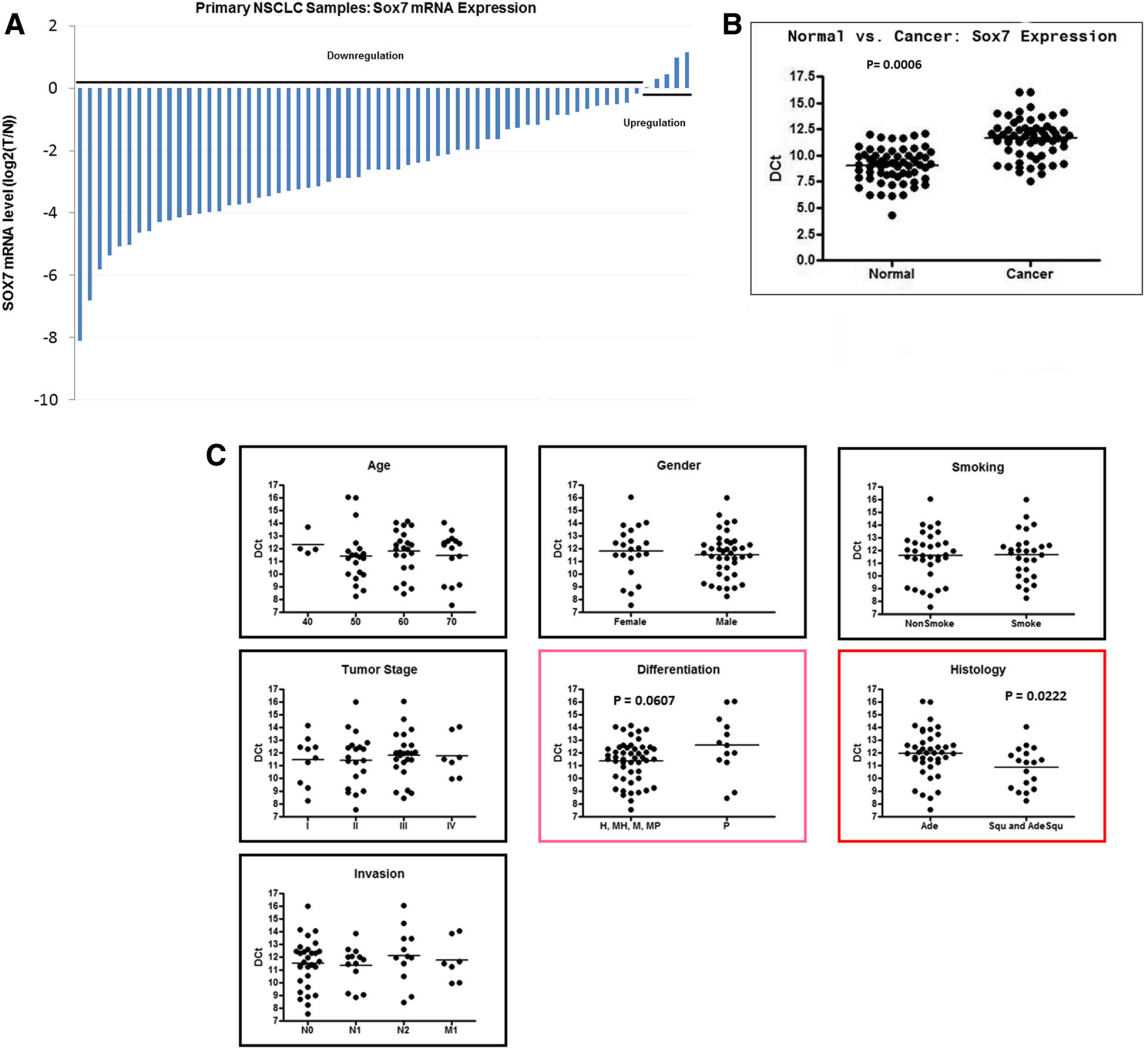
**Downregulated SOX7 in NSCLC compared to matched normal lung samples.** (**A**) Waterfall graph showing SOX7 mRNA expression in 62 paired human NSCLCs compared to normal lung tissue from the same patient. SOX7 mRNA expression was normalized to β-actin mRNA. (**B**) Statistical analysis of SOX7 mRNA expression in 62 paired human NSCLCs and normal lung tissues. Delta threshold cycle value (DCt) was calculated from the given threshold (Ct) value by the formula DCt = (Ct SOX7 – Ct β-actin) in each sample. P value was calculated with Paired *T*-test. (**C**) Relationship between significant SOX7 mRNA levels in the NSCLC samples and clinicopathological features of the patients and their NSCLC. Statistical differences were observed in histological differences (p=0.0222). The p values were calculated with Mann–Whitney *T*-test.

Next, a large number of clinical NSCLC samples were examined for expression levels of SOX7 mRNA in 62 pairs of tumors and their matched normal lung tissues using qRT-PCR (Figure 3A). Paired *T*-test analysis showed that the expression of SOX7 mRNA was significantly decreased in fifty-seven of 62 (92%) NSCLC samples compared with adjacent normal lung tissues (p= 0.0006) (Figure 3B). The correlation between SOX7 mRNA levels, and clinical as well as pathologic characteristics was analyzed (Figure 3C). Expression levels of SOX7 mRNA were correlated with histology (adenocarcinoma had lower expression than either squamous or adenosquamous carcinoma, p= 0.0222) and tumor differentiation (poorly differentiated had lowest expression, p= 0.0607). In contrast, no significant correlations were identified between SOX7 expression in the NSCLC and age, gender, smoking history, tumor stage and invasion (Figure 3C).

### Upstream region of SOX7 gene in lung cancer cell lines was highly methylated

**Figure 4:**
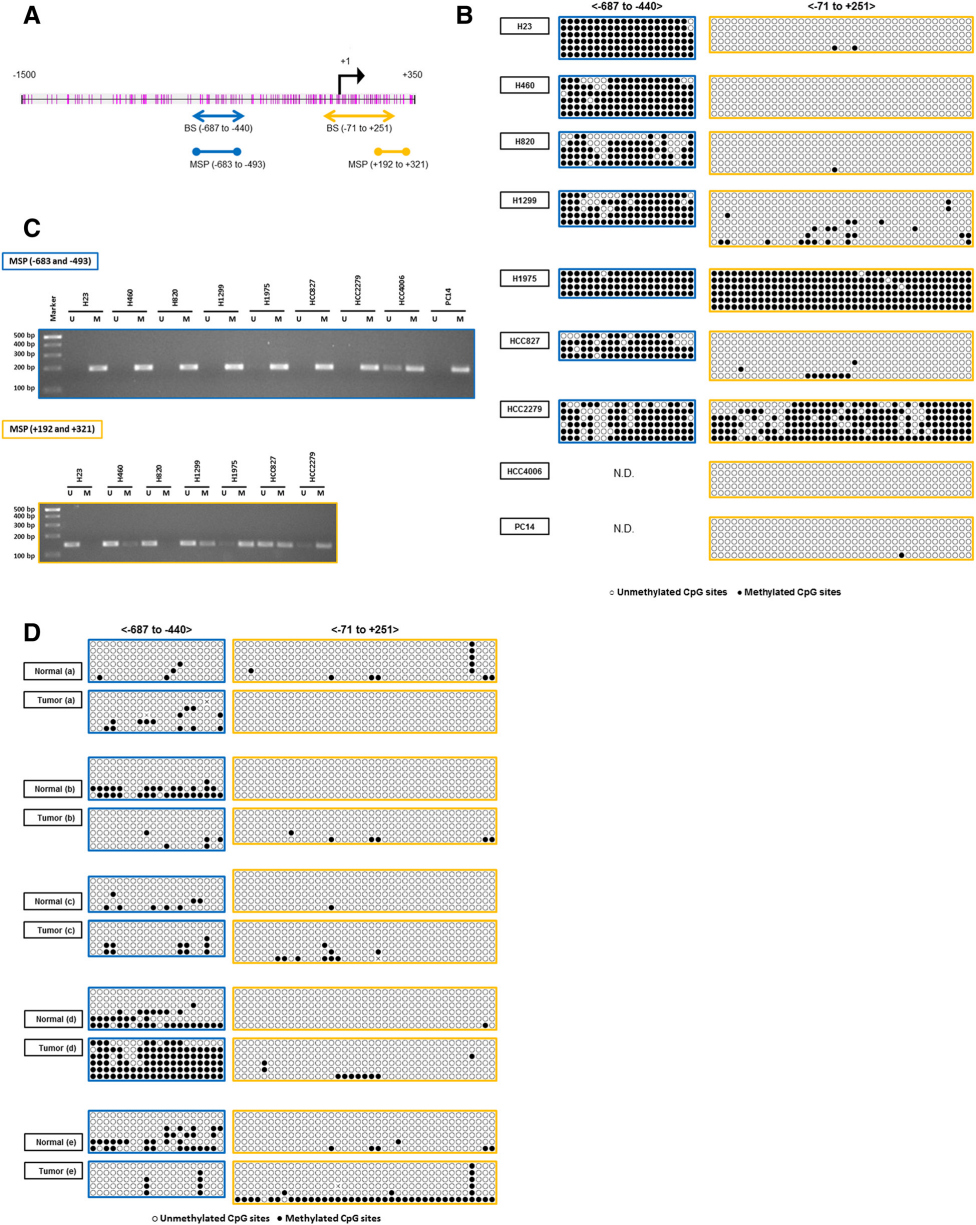
**Methylation analysis of upstream regions of SOX7 gene. **(**A**) Schematic illustration of CpG sites spanning 1,500 bp upstream and 350 bp downstream of the transcription start site of SOX7 transcription. Black arrow denotes the transcription start site (+1). Vertical pink bars denotes the CpG sites. Arrowed bars define the regions subjected to bisulfite sequencing (BS). Bars with circles at their end define the regions subjected to methylation specific PCR (MSP). (**B**) Bisulfite sequencing of SOX7 gene in NSCLC cell lines. Each circle in each horizontal row represent the analysis of a single clone of bisulfite-treated DNA encompassing either 20 or 35 CpG sites (-678 to -440, left panels; -71 to +251, right panels, respectively). Open and solid circles represent unmethylated and methylated CpG sites, respectively. (**C**) MPS analysis of upstream region of SOX7 gene in NSCLC cell lines. PCR products in lanes marked “U” and “M” are obtained with unmethylated-specific and methylated-specific primers, respectively. (**D**) Bisulfite sequencing of SOX7 gene in NSCLC samples and their matched normal lung tissues as described for the NSCLC cell lines in Figure 4B.

The mechanism underlying the down-regulation of SOX7 expression in lung cancer was explored. The upstream region of SOX7 gene has several dense CpG islands (Figure 4A). Primers for Bisulfite Sequencing and Methylation Specific PCR (MSP) assays were designed (Figure 4A). Bisulfite Sequencing analysis showed that the upstream CpG rich region (-687 to -440) was hypermethylated in all 7 of the examined NSCLC cell lines. The downstream region (-71 to +251) was hypermethylated in two (H1975 and HCC2279) of 9 NSCLC cell lines (Figure 4B). MSP analysis confirmed the Bisulfite Sequencing technique, showing that the upstream region (-683 to -493) was highly methylated in eight (H23, H460, H820, H1299, H1975, HCC827, HCC2279, PC14) of the 9 NSCLC cell lines (Figure 4C and Table 3). As expected, we could not amplify either the upstream or downstream regions of the SOX7 gene in the HCC2935 cells consistent with a homozygous deletion of the gene in these cells (data not shown). A perfect correlation between upstream methylation and SOX7 expression did not occur. HCC4006 had only modest positivity by MSP but did not express SOX7; and PC14 was methylated by MSP examination, but expressed SOX7. Also in contrast to the cell line data, the Bisulfite Sequencing analysis showed that the upstream region (-687 to -440) was hypermethylated in one of 5 lung tumor samples. We did not have RNA or protein available for these samples to examine SOX7 expression. The downstream region (-71 to +251) was neither methylated in NSCLC nor matched normal samples (Figure 4D), which was consistent with the methylation pattern noted in the NSCLC cell lines.

**Table 3:** Summary of methylation analysis of SOX7

**Cell Lines**	**SOX7 Western**	**BS (-687 and -493)**	**MSP (-683 and -493)**	**BS (-71 to +251)**	**MSP (+192 and +321)**
H23	-	(98%)	M	(<1%)	U
H460	+/-	(92%)	M	(0%)	U
H820	-	(70%)	M	(<1%)	U
H1299	-	(85%)	M	(8%)	U, M
H1975	-	(99%)	M	(99%)	U, M
HCC827	-	(80%)	M	(3%)	U, M
HCC2279	-	(75%)	M	(75%)	U, M
HCC2935	-	Deleted	Deleted	Deleted	Deleted
HCC4006	-	N.D.	U, M	(0%)	N.D.
PC14	++	N.D.	M	(<%)	N.D.

-, +/-, ++: no, slight, moderate SOX7 protein expression.

*BS*, Bisulfite sequencing; *MSP*, Methylation specific PCR; *U*, Unmethylated; *M*, Methylated; *N*.*D*., Not done.

### Forced-expression of SOX7 in NSCLC cells slowed their proliferation

**Figure 5:**
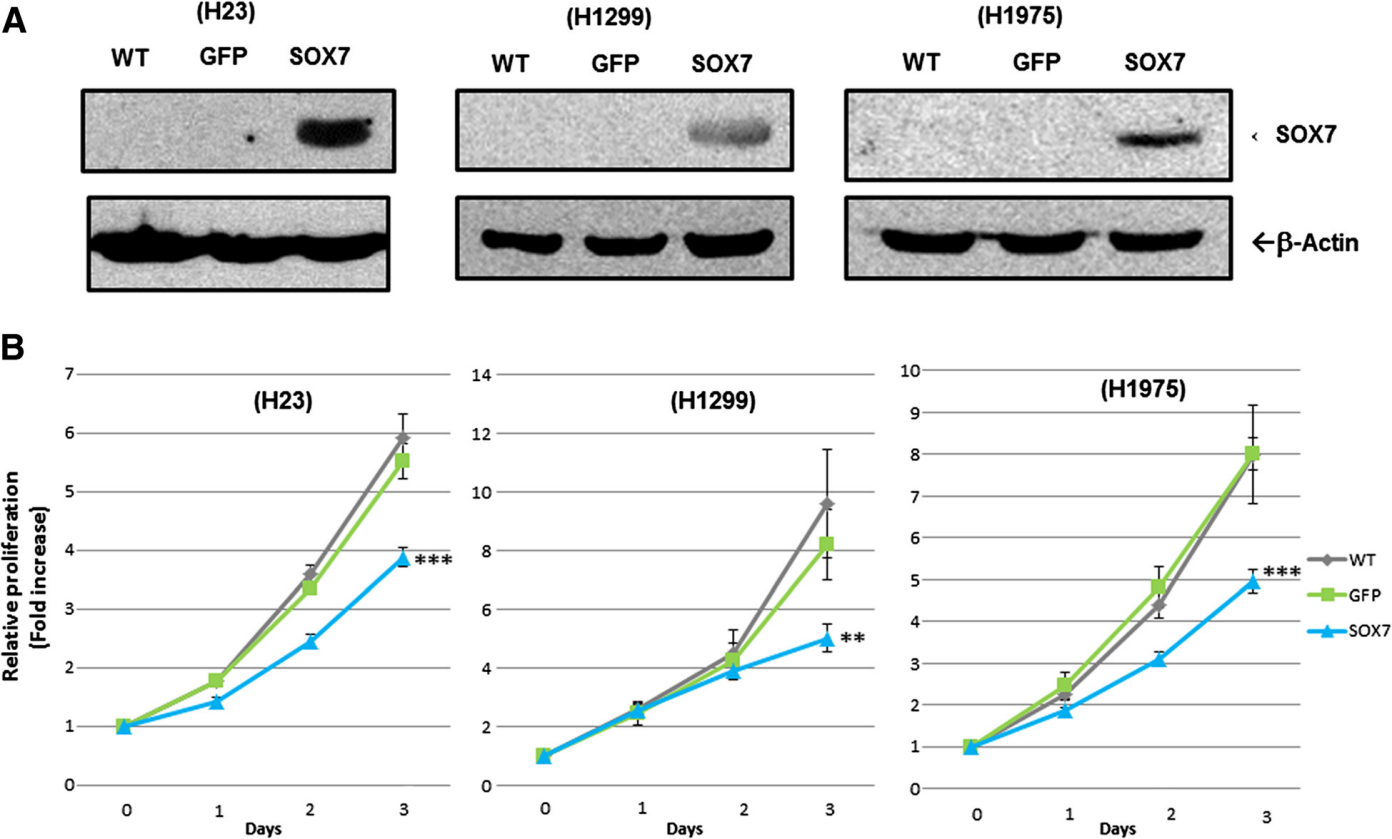
**Forced**-**expression of SOX7 slows NSCLC proliferation. **NSCLC cell lines (H23, H1299 and H1975) were stably infected and selected for stable expression of SOX7. (**A**) SOX7 vector uninfected (WT), GFP expression vector infected (GFP) or SOX7 expression vector infected SOX7 cells were confirmed in the three NSCLC cell lines by western blotting. β-actin was the control for equal loading. (**B**) Proliferation was measured by MTT assay. Each cell line was seeded in 96 well plates and absorbance was measured after 1, 2, 3 and 4 days culture. Results show the mean ±SD of quintuple wells. ** or ***, signifies statistical differences p < 0.01 or p < 0.001, respectively.

We developed stable clones of three NSCLC cell lines (H23, H1299, H1975) expressing a SOX7 expression vector (Figure 5A). These NSCLC cells had statistically significantly slower growth than the vector control cells (H23 and H1975, p= < 0.001 and H1299, P=<0.01, respectively) (Figure 5B).

### Effect of SOX7 expression on cell cycle regulation

**Figure 6:**
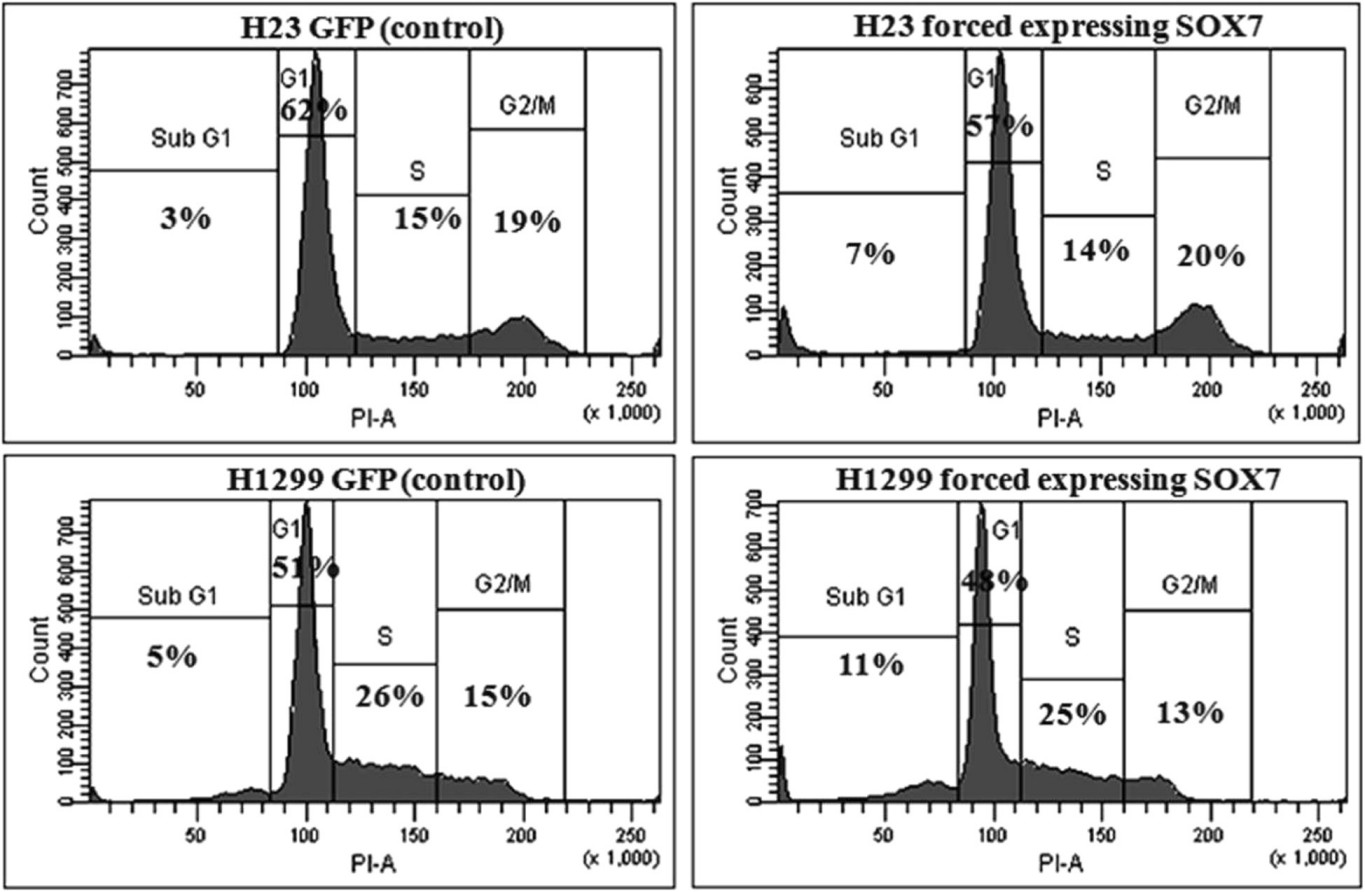
**Forced**-**expression of SOX7 increases subG1 phase of cell cycle in NSCLC.** Histogram represents the distributions of cells (H23 and H1299) in sub-G_1_, G_0_/G_1_, S and G_2_/M phases as determined by flow cytometry. Forced expression of SOX7 resulted in increased percentage of cells in subG1 phase of cell cycle in H23 and H1299 compared to GFP (control) cell. The figure is the representative of three independent experiments.

To study the effect of SOX7 expression on the cell cycle, we used H23 and H1299 human lung cancer cell lines stably expressing either SOX7 or GFP (used as control). Fluorescence-activated cell sorting (FACS) analysis for the cell cycle showed that forced expression of SOX7 in H23 and H1299 cell lines resulted in an accumulation of a sub-G1 peak compared to the control cells. The percentage increase in the sub-G1 phase was from 3% (control) to 7% (SOX7) for H23 cells and 5% (control) to 11% (SOX7) for H1299 cells. The proportions of cells in the other phases of the cell cycle were generally unchanged in experimental versus control cells. These results demonstrate that SOX7 forced expression in lung cancer cell lines was associated with a sub-G1 population which probably reflected apoptosis (Figure 6).

### Forced expression of SOX7 induces apoptosis in H23 and H1299 cell lines

**Figure 7:**
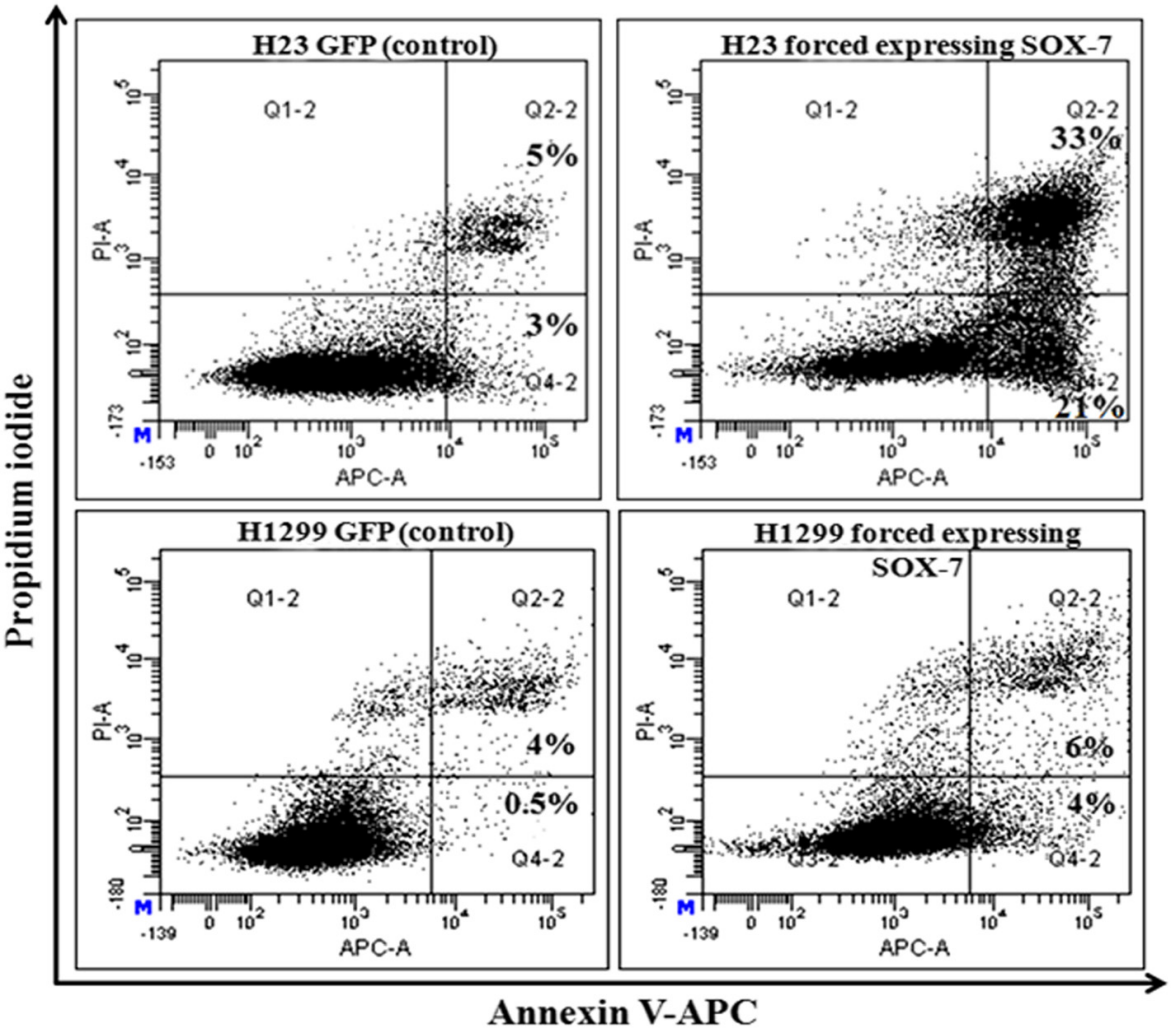
**Forced**-**expression of SOX7 increases apoptosis in NSCLC by Annexin V**-**PI staining. **Flow cytometry profile represents Annexin V-FITC staining in X-axis and propidium iodide in Y-axis. Dual staining of cells with Annexin V-APC and propidium iodide enabled categorization of cells into four regions. Region Q1 shows the necrotic cells, Q2 shows the late apoptotic cells, Q3 shows the live cells and Q4 shows the early apoptotic cells. Forced expression of SOX7 resulted in increase of early and late apoptotic cells in H23 and H1299 compared to GFP (control) cell. The figure is the representative of three independent experiments.

To explore further whether forced expression of SOX7 resulted in apoptotic cell death, Annexin V-APC/propidium iodide (PI) staining was performed for H23 and H1299 human lung cancer cell lines stably expressing either SOX7 or GFP (used as control). Based on Annexin V and PI staining, SOX7 expression led to increased early (AV^+^PI^-^), as well as, late (AV^+^PI^+^) apoptotic cells. A notable 21% and 33% of the H23 SOX7 cells were early and late apoptotic cells, respectively. In comparison, 3% and 5% of the H23 GFP cells (control cells) were early and late apoptotic cells, respectively. Less dramatically, 4% and 6% of early and late apoptotic H1299 SOX7 cells, respectively compared to 0.5% and 4% of early and late apoptotic H1299 GFP cells (control), respectively (Figure 7).

## Discussion

We initially performed CN analysis of 9 NSCLC samples and 8 NSCLC cell lines, each with an EGFR mutation. Their pattern of genome alterations were compared to the SNP-Chip copy number changes found in 56 NSCLC in the TCGA data base. Our samples were from non-smoking Asians who had EGFR mutations. The TCGA samples were composed of predominantly Caucasians who smoked and therefore less than 7% of samples would be expected to contain an EGFR mutation [14]. Remarkably, their genomic landscape of copy number change was very similar. All the samples had increase in CN throughout the genome (predominantly 3N), especially at 1q, 5p, 7p, 8q, 11q, 12q, 14q, 17q. However, although sample numbers were small, eight genome regions had notable difference in copy number changes between the NSCLC samples with EGFR mutation compared to those in the TCGA data base samples (Table 2) including 1p36.31-36.32 [8/9 (89%) versus 15/56 (27%)] and 19q21.3, [5/9 (56%) versus 6/56 (11%)], respectively. Further studies are required to clarify what the target genes are in these regions (Table 1).

One of the NSCLC cell lines (HCC2935) had a homozygous mutation at 8p23.1 which encompassed the SOX7 gene (Figure 1). Interestingly, 8p is one of the few regions in the NSCLC samples associated with deletions. Homozygous deletion usually represents the loss of a tumor suppressor gene deleted by the tumor. Our further studies focused on SOX7. Expression levels of SOX7 mRNA and protein were diminished in eight of 10 NSCLC cell lines (Figure 2), as well as in fifty-seven of 62 (92%) NSCLC patient samples compared with their matched normal tissues. Expression level of SOX7 in NSCLC samples was correlated with their histology, with levels being lower in adenocarcinomas compared with adenosquamous and squamous carcinomas (Figure 3). Furthermore, force-expression of SOX7 in several NSCLC lines (H23, H1299, and H1975) having constitutively low level of SOX7, suppressed their cellular proliferation and enhanced their apoptosis (tested with H23and H1299) (Figure 5, 6 and 7).

Recent studies of SOX7 in colorectal and prostate cancers showed that levels of this transcription factor were low in these cancers in part due to aberrant DNA methylation of the gene, and the protein behaved as a tumor suppressor gene in these cancers [10,15]. We found that the upstream region (-687 to -440) of SOX7 was highly methylated in eight of 10 NSCLC cell lines (Table 3). Paradoxically, expression of SOX7 and methylation as measured by MSP analysis were not correlated in the H460 and PC14 cells, and only one of 5 fresh NSCLC samples was highly methylated in the promoter region of SOX7. This suggests that additional epigenetic changes are required for silencing of this gene in a proportion of NSCLC.

In summary, our study suggests that SOX7 is a tumor suppressor in the lung. One or occasionally both alleles are lost in the lung cancer. Other times the upstream CpG island of the SOX7 gene is robustly methylated, associated with low expression of the gene. SOX7 levels were nearly undetectable in seven of 9 (78%) highly methylated NSCLC cell lines, and levels were low in 57 of 62 (92%) NSCLC samples compared to adjacent normal tissues. Loss of SOX7 expression appears to provide a growth advantage to NSCLC cells.

## Competing interests

The authors declare that they have no competing interests.

## Authors’ contributions

TH, MG and DY conceived and designed the study, performed the interpretation of data, literature search and writing. MS, NK, SS, WC and LWD performed statistical analysis and data interpretation. GL carried out analysis and writing. SM and DX performed patient collection and clinical data interpretation. PT participated in the study design. HPK conceived and designed the study, carried out data interpretation and analysis, and writing. All authors read and approved the final manuscript.
